# Chemotherapy related encephalopathy in a patient with Stage IV cervical carcinoma treated with cisplatin and 5-fluorouracil: a case report

**DOI:** 10.4076/1757-1626-2-8526

**Published:** 2009-07-30

**Authors:** Amy L Chue, Indrajit N Fernando, Syed A Hussain, David A Yates

**Affiliations:** 1 Oncology Department, Queen Elizabeth Hospital, University Hospital Birmingham NHS TrustEdgbaston, West MidlandsUK; 2 Neuroradiology Department, Queen Elizabeth Hospital, University Hospital Birmingham NHS TrustEdgbaston, West MidlandsUK

## Abstract

**Introduction:**

Chemotherapy related encephalopathy is commonly reported with certain forms of chemotherapy but few reports note an association with low dose 5-Fluorouracil.

**Case presentation:**

A 57-year-old Caucasian lady received her first cycle of Cisplatin and 5-Fluorouracil for palliative treatment of cervical carcinoma, and presented several days later with signs of encephalopathy. Several causes were eliminated, and encephalopathy related to 5-Fluorouracil was thought to be the most likely cause. Magnetic Resonance Imaging of the head revealed changes related to the chemotherapy received. Symptoms resolved completely within three days of presentation.

**Conclusion:**

Encephalopathy from low dose 5-Fluorouracil is not well documented in the literature. Fluid rehydration and supportive treatment is required. Signs and symptoms resolved completely with no residual effects on follow up.

## Introduction

Certain types of chemotherapy are more commonly associated with neurotoxic side effects, most commonly Ifosfamide and Methotrexate [1]. High doses of 5-Fluorouracil have been reported to cause neurological signs and symptoms, but only a few cases of encephalopathy have been recorded when low doses, or boluses, of 5-Fluorouracil have been used [2,3].

Fluorouracil is an analogue of fluorinated pyramidine commonly used in the treatment of several cancers. After rapid intravenous injection, fluorouracil rapidly diffuses into all body compartments, including the nervous system. The drug is primarily degraded by the liver. The toxicity of 5-FU is strongly influenced by the dosage used and the rate and duration of drug administration. Neurological toxicity manifested by somnolence, confusion, seizures, cerebellar ataxia and rarely encephalopathy are known but uncommon. They are usually totally reversible on withdrawal of the drug. Leucovorin which is commonly combined with 5-FU enhances the antitumour activity as well as toxicity [5].

## Case presentation

A 57-year-old Caucasian lady was admitted to hospital feeling generally unwell with nausea, lethargy and reduced appetite. She had been unwell since discharge from hospital two weeks ago after her first in-patient cycle of Cisplatin and 5-Fluorouracil (5-FU). She was diagnosed with FIGO (International Federation of Gynaecologists and Obstetricians) Stage IVb mucinous adenocarcinoma of the endocervix seven months previously, an incidental finding after a laparotomy and hysterectomy were performed for abdominal distension. No other metastases were found at the time, but a relapse in the vaginal vault and small bowel were identified on repeat laparotomy. She proceeded to external beam radiotherapy (45 Gy/25 Fractions over five weeks) to the pelvis with concurrent Cisplatin, 40 mg/sqm. During this period of treatment, she developed a thrombus in the inferior mesenteric vein and was treated with low molecular weight heparin. Blood counts and biochemical profile were normal throughout chemoradiation. In-patient chemotherapy was started one month after completing radiotherapy, consisting of Cisplatin (75 mg/sqm) and 5-FU (1 g/sqm), as a four day cycle.

A past medical history of Peutz-Jegher’s syndrome, diagnosed at the age of 17, resulted in three bowel operations over two decades, leaving her with an ileostomy. She also had a history of breast cancer 25 years ago, for which a left mastectomy, axillary node sampling and a bilateral oophorectomy were performed. No further radiotherapy, chemotherapy or Tamoxifen was given. Drug history was insignificant.

She presented to hospital two weeks after the first cycle of Cisplatin and 5-FU, feeling unwell. She was dehydrated with renal impairment; urea was 12.8 mmol/l and creatinine 154 mmol/l. Other biochemical markers, including calcium, potassium and magnesium were normal, and C-reactive protein (CRP) was 3 mg/l, haemoglobin 10.5 g/dL, Sodium 141 mmol/l.

Rehydration continued over the next few days. She remained apyrexial with oxygen saturations of 98-100% on room air.

Three days after admission, the patient described an episode of brief confusion in the morning. Renal function was improving and biochemistry remained normal with a CRP of 4 mg/l. Later the same day and into the early hours of the morning, her confusion returned and she became incontinent of urine. She no longer communicated with staff verbally, and repetitive actions, such as licking her lips and moving her hand round in circles, were observed. She showed no aggressive or disruptive behaviour, but refused to have bloods taken or a cannula inserted by pushing staff away. No prior mental health issues were noted in hospital records or from family.

She was no longer orientated in time, place or person. Her abbreviated mental test score (AMTS) was 0 out of 10 and repetitive movements of her right arm continued. Her temperature was 37°C, chest remained clear on examination, abdomen soft with bowel sounds present. Glasgow Coma Score (GCS) was 14/15. Random blood glucose was within normal limits and urine dipstick was negative. Blood tests were repeated and sent for full blood count, biochemical profile, CRP and blood culture. A CT head was booked urgently and she was commenced on Dexamethasone 4 mg twice a day.

Night review by the on-call doctor found the patient groaning and making incomprehensible sounds and thrashing around. Repetitive movements continued but no seizure activity was noted. Midazolam and Lorazepam were given with minimal benefit.

Biochemistry remained normal, blood cultures were negative, with a normal thyroid stimulating hormone (TSH), normal calcium, potassium and magnesium and CRP of 8 mg/l, haemoglobin 10.6 g/dL, white cell count 6.1 × 109/l, sodium 143 mmol/l. Autoantibody screen was negative and electrophoresis showed no qualitative abnormality.

CT scan of the head with contrast was conducted, which reported no sign of intra- or extra-cranial abnormality.

The following day, day six of admission, the patient was found to be smiling and lucid. She was orientated in time, place and person. The Neurology team were asked to advise and their differential diagnosis consisted of non convulsive status, acute mal encephalitis, paraneoplastic limbic encephalitis, drug related encephalopathy, sinus vein thrombosis or infectious encephalitis. They suggested the following further investigations; MRI brain, to exclude temporal lobe inflammation, lumbar puncture, to assess for infection or neoplastic infiltration, and an EEG.

The patient was much improved. She was eating and drinking, communicating normally with staff and her husband, with no obvious immediate adverse effect of the acute confusional state. Her renal function remained impaired, but all other bloods were normal.

Unfortunately, patient consent could not be obtained for a lumbar puncture or an EEG, despite emphasis given to their importance. At the time of asking, the patient had improved and was deemed to have capacity.

**Figure 1.**
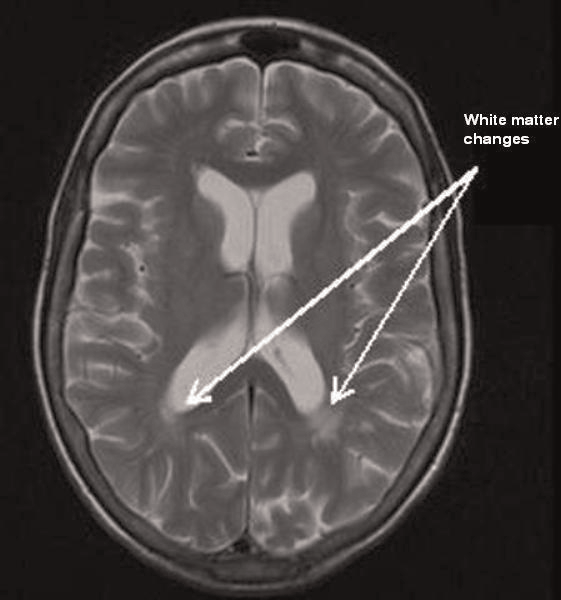
Magnetic Resonance Imaging showing white matter changes in the parietal area post gadolinium enhancement.

**Figure 2.**
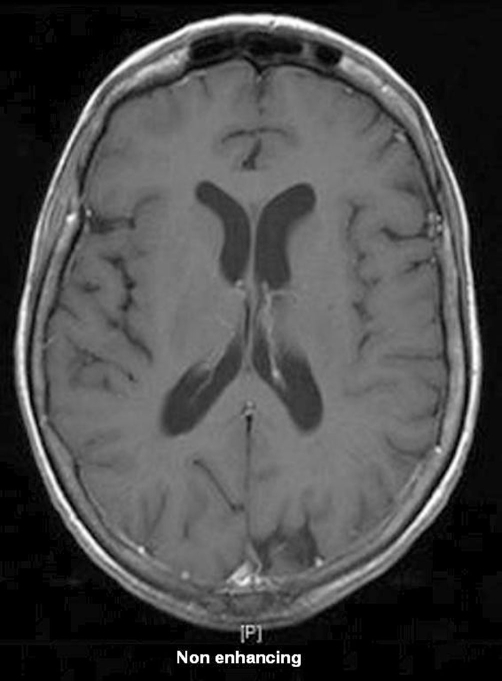
Magnetic Resonance Imaging showing nothing remarkable pre enhancement.

The MRI concluded that there were small amounts of non-enhancing white matter change in the parietal lobes (Figure 1 and Figure 2).

## Discussion

Given the unlikelihood of metastatic disease, sepsis or a severe metabolic disorder causing the acute confusion, we assessed the likelihood of the events being caused by chemotherapy, in particular, 5-FU.

Several cases of chemotherapy related encephalopathy are noted in the literature, with some agents being more problematic than others. Neurotoxicity is a major limitation of many drugs used in cancer patients, and as more and more chemotherapy agents are being added to the list, physicians must be wary of any signs of neurotoxicity after chemotherapy cycles [1]. Magnetic resonance imaging makes recognition of central nervous system toxicity easier [4].

Ifosfamide related encephalopathy is well known [1], occurring in 15-30% of patients treated with the chemotherapy agent. Symptoms of encephalopathy can arise soon after receiving combination chemotherapy involving Ifosfamide for different tumours [5]. Patients often recover after discontinuation of the agent. The administration of methylene blue (methylthioninium chloride) has little evidence as an antidote [6].

Another chemotherapy agent of interest is Methotrexate, which forms the mainstay of treatment for patients with osteosarcoma. It is also a major cause of treatment related acute neurotoxicity, especially at the high doses which it is prescribed [1]. Cases have been noted to cause white matter injury on MRI at the time of symptoms, which do not show contrast enhancement. Such injuries appear to resolve on follow up scans [7].

The significance of hyperammonaemic encephalopathy as a cause for neurological deterioration post chemotherapy has also been reported. Characteristics include sudden alteration in mental status accompanied by markedly elevated plasma ammonia levels in the absence of obvious liver disease or any other identifiable cause. More importantly, it is known to occur in solid organ malignancies treated with 5-Fluorouracil [2,8].

Cisplatin has also been noted to cause neurological symptoms, most commonly a peripheral sensory neuropathy; however, encephalopathy and seizures have also been observed, especially following rechallenge with the Platinum agent [9,10]. More commonly however, toxicity is related to hypomagnesaemia and hypokalaemia and in cases of rapid increases in blood pressure, such as renal disease or hypertensive encephalopathy, where such conditions can exacerbate the ensuing encephalopathy [11]. Doses of Cisplatin must be adjusted in cases of renal failure due to cumulative nephrotoxicity. In this case, consideration must be given to the impaired renal function which may have contributed to the neurological symptoms by enhancing the effects of Cisplatin; however, renal function at the time of administration of chemoradiotherapy and the first cycle of Cisplatin/5-FU was normal, and given no previous side effects or nephrotoxicity were observed with Cisplatin when given with concurrent radiotherapy for five weeks, it is unlikely that the renal impairment or the neurotoxicity were due to Cisplatin.

Cases have commonly reported high dose 5-FU induced encephalopathy, [12,13] but encephalopathy associated with lower doses is less common. Our patient received a total of 4000 mg/sqm over a four day period, 1000 mg/sqm daily, a relatively low dose of 5-FU. In comparison, several case reports have linked symptoms of encephalopathy with daily high doses of 1500 mg or higher. Reported cases of low dose encephalopathy are rare. The few that have been reported note the absence of structural abnormalities, but do show an association with hyperammonaemia in specific cases [2,3]. The significance of the raised ammonia level is yet to be uncovered. Patients present with encephalopathy, including confusion, cognitive dysfunction, disorientation, agitation, lethargy, seizure, cerebellar ataxia, nystagmus and coma [2,3]. Diagnostic criteria include 1. Development of encephalopathy during or shortly after completion of 5-FU therapy; 2. Exclusion of other metabolic or physical factors that may effect level of consciousness; 3. Exclusion of drug effect by concomitant medications [3]. An important point to make in such a situation, is the possibility of a deficit in dihydropyrimidine dehydrogenase (DPD). DPD is the initial and rate-limiting enzyme in the catabolism of 5-FU. More than 80% of administered 5-FU is eliminated by DPD, therefore implying that a deficiency in the enzyme would result in highly toxic effects such as severe mucositis, myelosuppression/neutropenia and severe diarrhoea. Encephalopathy and hepatitis are noted to be rarer manifestations of toxicity as a result of enzyme deficiency. Only one of the reported cases of 5-FU induced encephalopathy measured DPD levels using PCR, which increased after administration of 5-FU, therefore suggesting an unlikely DPD deficiency [2]. It is thought that DPD deficiency in our patient is unlikely, given the lack of any of the other severe toxic effects such as mucositis or neutropenia being present. Malnutrition and thiamine deficiency are also thought to contribute to this neurological syndrome [3].

## Conclusion

Few cases have been documented, but in most, neurological features are transient and recovery often complete. This patient made a complete recovery after the solitary episode of confusion, and is doing well with no residual symptoms three weeks on from presentation. Given her reaction to chemotherapy, a decision was taken to not attempt a rechallenge of 5-Fluorouracil in this patient. Recognition of such cases is imperative in order to maximise treatment after exclusion of all other possible causes.

## Abbreviations

AMTS: Abbreviated Mental Test Score
CT head: Computer Tomography of the head
CRP: C-reactive protein
DPD: Dihydropyrimidine dehydrogenase
EEG: Electroencephalogram
FIGO: International Federation of Gynaecologists and Obstetricians
GCS: Glasgow Coma Scale
Gy: Gray - dose of Radiotherapy
mg: milligrams
mg/sqm: milligrams per metre squared
mmol/l: millimoles per litre
MRI: Magnetic Resonance Imaging
PCR: Polymerase Chain Reaction
TSH: Thyroid Stimulating Hormone
5FU: 5-Fluorouracil
